# How Far into Europe Did Pikas (Lagomorpha: Ochotonidae) Go during the Pleistocene? New Evidence from Central Iberia

**DOI:** 10.1371/journal.pone.0140513

**Published:** 2015-11-04

**Authors:** César Laplana, Paloma Sevilla, Juan Luis Arsuaga, Mari Carmen Arriaza, Enrique Baquedano, Alfredo Pérez-González, Nieves López-Martínez

**Affiliations:** 1 Museo Arqueológico Regional de la Comunidad de Madrid, Alcalá de Henares, Madrid, Spain; 2 Departamento de Paleontología, Facultad de Ciencias Geológicas, Universidad Complutense de Madrid, Madrid, Spain; 3 Centro Mixto UCM-ISCIII de Evolución y Comportamiento Humanos, Madrid, Spain; 4 Departamento de Geología, Geografía y Medio Ambiente, Universidad de Alcalá, Alcalá de Henares, Spain; 5 Instituto de Evolución en África (IDEA), Madrid, Spain; 6 Centro Nacional de Investigación sobre la Evolución Humana (CENIEH), Burgos, Spain; Universita degli Studi di Roma La Sapienza, ITALY

## Abstract

This paper reports the first find of pika remains in the Iberian Peninsula, at a site in central Spain. A fragmented mandible of *Ochotona* cf. *pusilla* was unearthed from Layer 3 (deposited some 63.4±5.5 ka ago as determined by thermoluminescence) of the Buena Pinta Cave. This record establishes new limits for the genus geographic distribution during the Pleistocene, shifting the previous edge of its known range southwest by some 500 km. It also supports the idea that, even though Europe’s alpine mountain ranges represented a barrier that prevented the dispersal into the south to this and other taxa of small mammals from central and eastern Europe, they were crossed or circumvented at the coldest time intervals of the end of the Middle Pleistocene and of the Late Pleistocene. During those periods both the reduction of the forest cover and the emersion of large areas of the continental shelf due to the drop of the sea level probably provided these species a way to surpass this barrier. The pika mandible was found accompanying the remains of other small mammals adapted to cold climates, indicating the presence of steppe environments in central Iberia during the Late Pleistocene.

## Introduction

It is well known that the succession of glacial and interglacial periods of the Pleistocene had a decisive impact on the distribution of mammals [1–5]. The pulsating expansion of the tundra-steppe biome well into Europe during the cold phases of the Pleistocene gave some species the opportunity to expand their previous distributions from central Asia into Europe. Such was the case of pikas (genus *Ochotona*), a group of small lagomorphs belonging to the family Ochotonidae. Pikas comprise 30 extant species mostly distributed in Asia, except for two species which can be found in North America [6]. According to their ecological preferences, two different groups of pikas can be recognized, rock-dwelling and meadow or steppe-dwelling [6].

The fossil record of *Ochotona* in Europe comprises at least three fossil taxa [7], recorded between the Late Pliocene and Early Pleistocene, as well as Middle Pleistocene to Recent fossil occurrences assigned to the living species *Ochotona pusilla* [8]. This last species, known as the steppe pika, was the mostly widespread pika during the Pleistocene, in particular during the cold phases of the Late Pleistocene when it extended its range into western Europe, reaching as far as the British Isles and the northern slopes of the Pyrenees [8–12]. Until now, no pika fossils were known from the Iberian Peninsula. This paper reports the first record for a pika in Spain found in the Buena Pinta Cave at Pinilla del Valle (Madrid Region). The find represents the currently known furthest expansion of the genus towards the southwest in its entire evolutionary history–an important record to accompany the few known for the genus in the peninsulas of the Mediterranean.

## Geographic and Geological Setting

**Fig 1:**
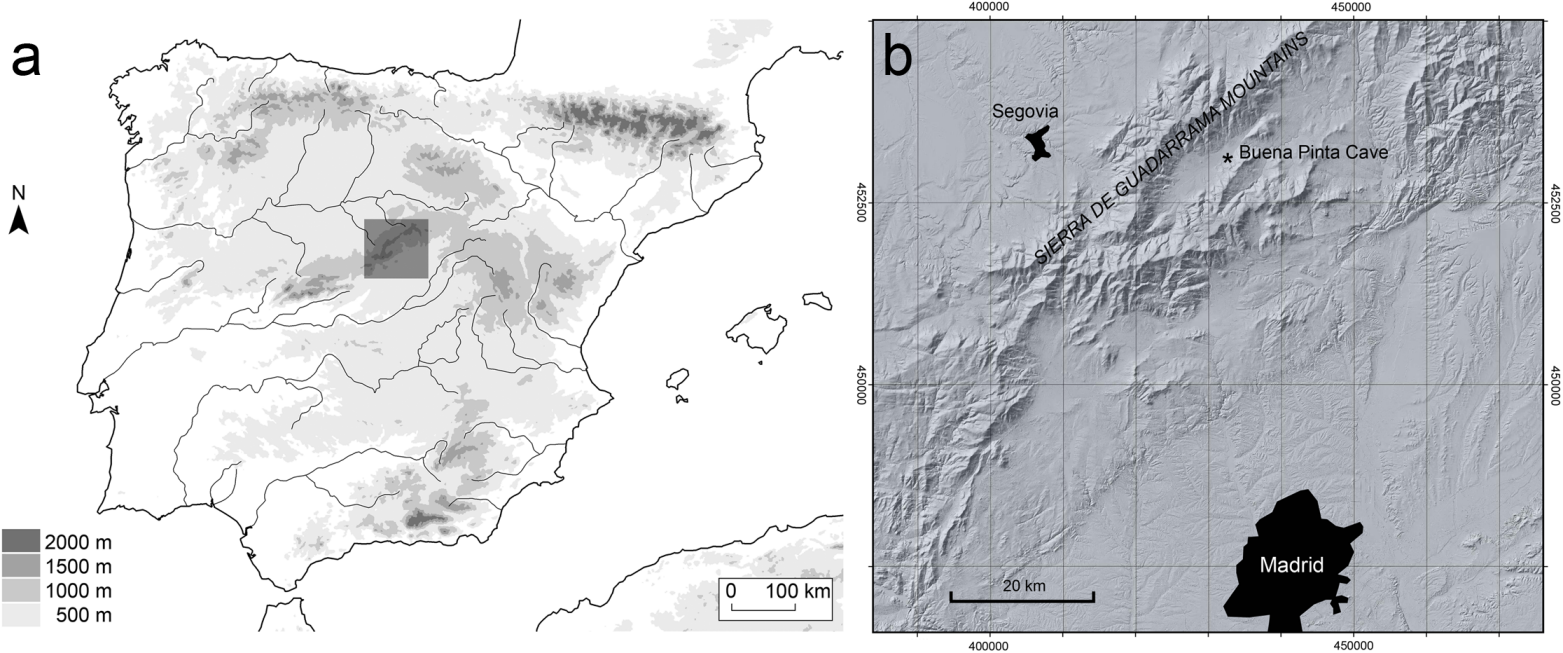
Location of the Buena Pinta Cave. **(**A) Map of the Iberian Peninsula, the dark rectangle marks the area enlarged in Fig 1B. (B) Digital terrain model of the area surrounding the site, with indication of the situation of Buena Pinta Cave.

The Buena Pinta Cave (one of the Calvero de la Higuera sites) is situated in the narrow Lozoya (or Paular) High Valley. Located 55 km north of Madrid, this SW-NE oriented valley occupies some 300 km^2^ within the Spanish Central System (the Sierra de Guadarrama Mountains; Fig 1). The valley floor lies at an altitude of 1000–1100 m. The enclosing peaks can reach over 2000 m; the highest is the Pico de Peñalara (2428 m) at the valley’s southwestern end. The pronounced altitudinal gradient and the area’s abundant rainfall together allow for a wide diversity of habitats, and consequently a rich flora and fauna. Indeed, the valley has 41 mammalian species (leaving out domestic forms and species that have become extinct in historic times through human action) [13], a figure distinctly higher than the mean for continental Spain [14]. Such exceptional ecological characteristics led the head of the Lozoya Valley to be declared a Natural Park in 1990, and recently in 2013 it was included in the Sierra de Guadarrama National Park. Similar high species richness occurred in the Middle and Late Pleistocene in this valley, as evidence by the fossil record preserved in the Calvero de la Higuera sites [15,16].

The Buena Pinta Cave was discovered in 2003 while conducting a survey in the area as part of a project to search for the earliest human settlements in central Iberia [17]. This small cave lies slightly above the bottom of the valley at an altitude of 1105 m. Its geographic coordinates are 40° 55’ 23.2” N, 3° 48’ 29.7” W (datum WGS84). It is completely filled with sediments of complex stratigraphy, and connected to the nearby Des-Cubierta Cave system (another site of karstic origin) through an equally sediment-filled gallery.

**Fig 2:**
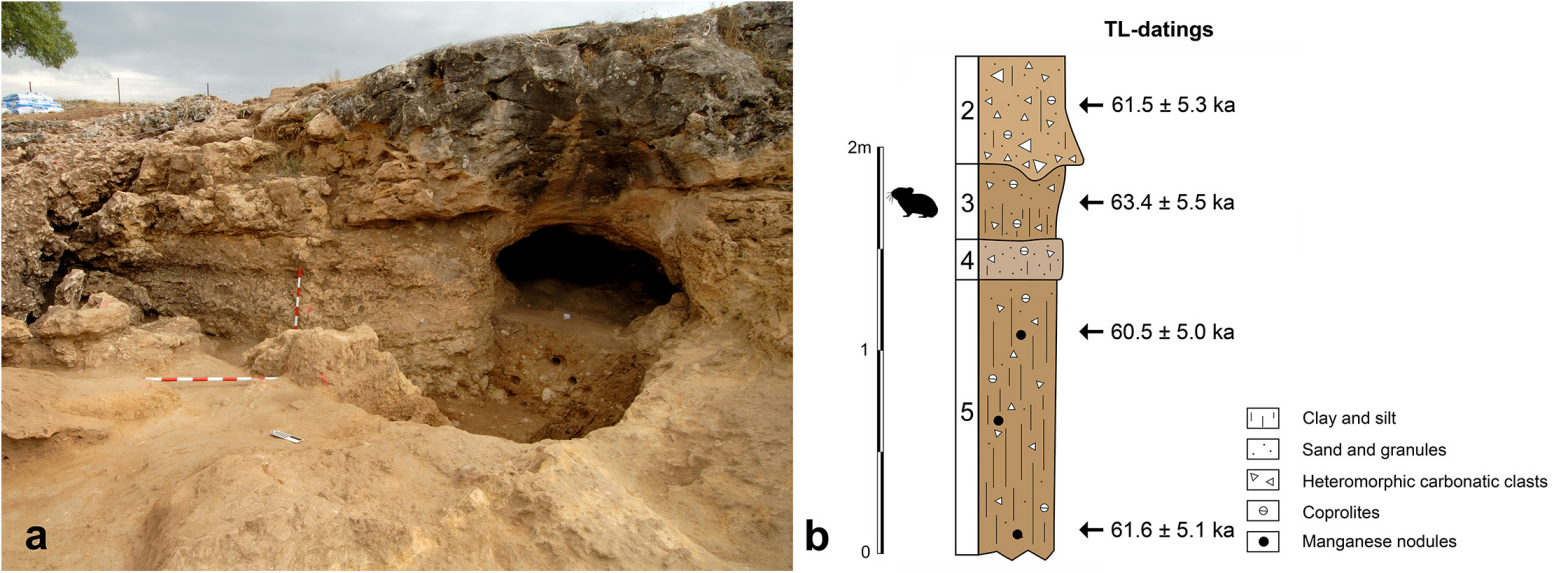
Stratigraphy and datings of the Buena Pinta Cave. (A) Picture of the current area of excavations at the external chamber of the cave and at the entry to the gallery. (B) Stratigraphy of the external chamber of the cave, with indication of the provenance of the samples of sediment dated by thermoluminiscence, and resulting datings (Dating and Radiochemistry Laboratory, Autonomous University of Madrid). The silhouette of a pika indicates the layer where the mandible of *O*. cf. *pusilla* was identified.

The Buena Pinta Cave sediments appear as three distinct units [18]. The upper unit (Layer 1), consists of clay and sand sediments up to 1.80 m thick; these are greyish in colour and contain carbonate clasts. The C14 AMS (2 sigma) values for this unit range from 5740–5610 years cal. BP at the base, to 1940–1800 years cal. BP at the top [19]. Underneath lies a set of sediments at least 2 m thick, consisting of a mixture of orange coloured sands, silt and clay with occasional carbonate clasts. Four approximately horizontal stratigraphic layers have been recognised in this unit, numbered 2–5 from top to bottom (Fig 2). Thermoluminescence dating performed on a sediment sample from Layer 3 has returned an age of 63.4±5.5 ka, placing this set of layers in the middle of the Late Pleistocene, within Marine Isotope Stage (MIS) 4 or the beginning of MIS 3 [18]. At the northwestern wall of the site, clast-supported conglomerates and bone breccias outcrop, which represent the oldest filling of the cavity. Particularly, according to its rodent taxa content (e.g., *Microtus brecciensis* and *Microtus vaufreyi*; [20]), the age of the northern wall can be dated to the second half of the Middle Pleistocene.

All the layers of this site are rich in bone remains of both large and small vertebrates. The pika remain described in this paper comes from Layer 3, where it formed part of a rich assemblage containing at least 34 small mammal species: *Arvicola sapidus*, *Arvicola amphibius s*. *l*., *Microtus arvalis*, *Microtus agrestis*, *Microtus cabrerae*, *Microtus* sp. gr. *M*. *duodecimcostatus-M*. *lusitanicus*, *Microtus oeconomus*, *Microtus gregalis*, *Chionomys nivalis*, *Myodes glareolus*, *Pliomys coronensis (= P*. *lenki)*, *Apodemus* gr. *sylvaticus-flavicollis*, *Allocricetus bursae*, *Eliomys quercinus*, *Sciurus vulgaris*, *Marmota marmota*, *Castor fiber*, *Oryctolagus cuniculus*, *Lepus* sp., *Erinaceus europaeus*, *Erinaceus* sp., *Sorex minutus*, *Sorex granarius*, *Neomys anomalus*, *Crocidura* sp., *Galemys pyrenaicus*, *Talpa occidentalis*, *Talpa europaea*, *Miniopterus schreibersii*, *Myotis* sp., *Myotis myotis/oxygnathus*, *Rhinolophus ferrumequinum*, *Rhinolophus hipposideros*, and *Rhinolophus euryale/mehelyi* [16,21]. *Microtus arvalis* dominates the assemblage, followed by *Microtus* sp. gr. *M*. *duodecimcostatus-M*. *lusitanicus* and *Microtus* gr. *agrestis*; indeed, these three vole species account for some 80% of the small mammal fossils collected from this layer. In addition to these rodents, the layer also contains darker-coloured (probably manganese oxide-stained) remains of the fossil taxa *M*. *brecciensis* and *M*. *vaufreyi* reworked from the breccias and conglomerates of the northern wall (the oldest unit). These species appear in low proportion (<1%) in this layer, but in the lower part of Layer 5 they reach proportions of 10–40% [16].

The number of small mammal species represented in Layer 3 makes it one of the largest assemblages described for a Late Pleistocene site in Iberia. Similar numbers have only been reported for a few sites of similar age in the Cantabrian region (one of the richest ecological regions of the Iberian Peninsula) [22]. The three vole species (*M*. *arvalis*, *M*. sp. gr. *M*. *duodecimcostatus-M*. *lusitanicus* and *M*. gr. *agrestis*) dominating the assemblage indicate that, during the formation of Layer 3, the landscape was mainly one of open spaces and grassland. This interpretation is also supported by the presence in the assemblage of other vole species (*M*. *gregalis*, *M*. *oeconomus*), the marmot, and the extinct hamster *A*. *bursae*. However, patches of forest must also have existed near the cave, as shown by the presence of a few *My*. *glareolus* and squirrel remains. Futhermore, the available pollen data [19] also indicate a cold, dry environment dominated by grassland.

## Material: Description and Identification

**Fig 3:**
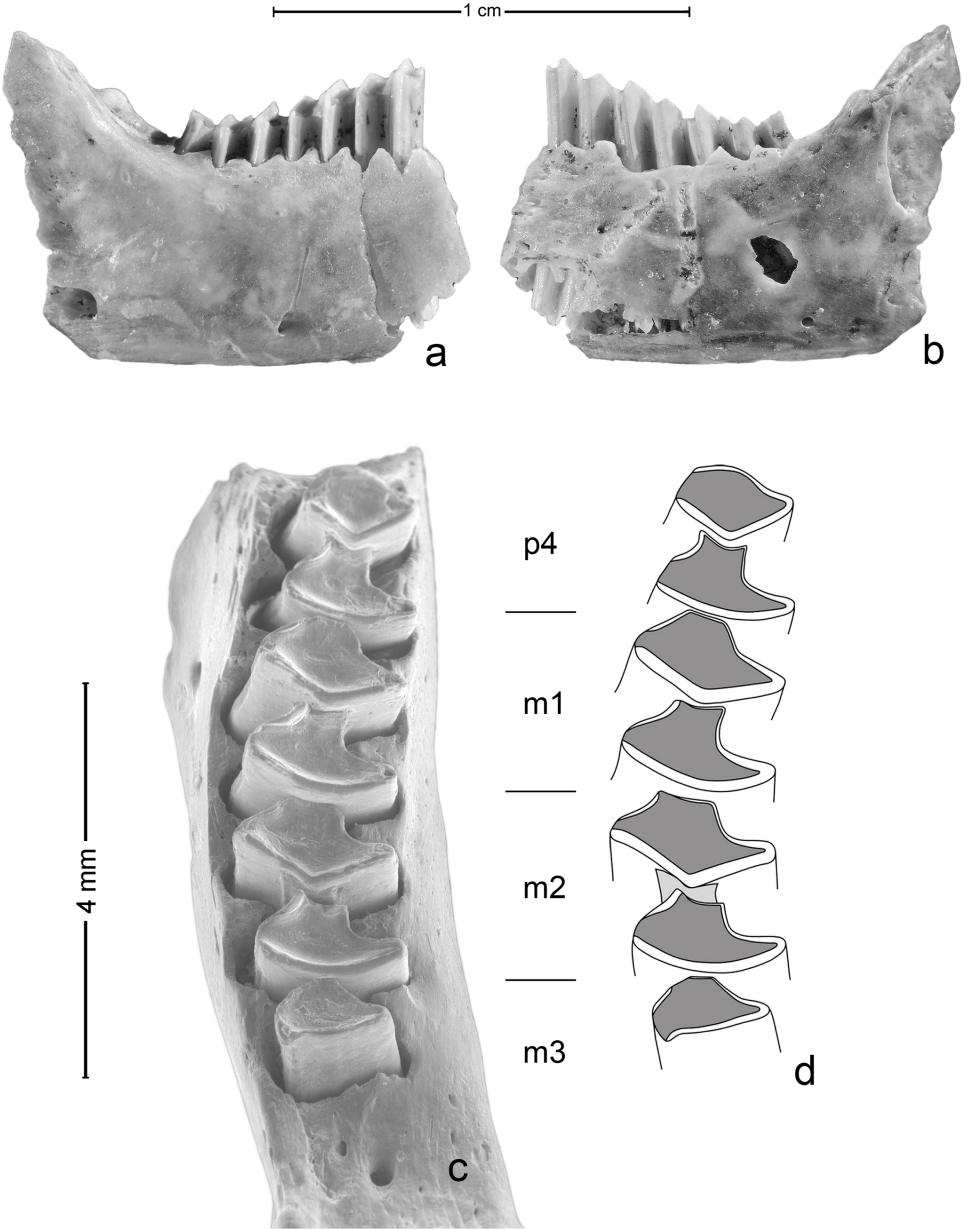
Fragment of a mandible from *Ochotona* cf. *pusilla* from Layer 3 of the Buena Pinta Cave. (A) Buccal view. (B) Lingual view. (C) SEM image of the teeth in occlusal view. (D) Sketch of the tooth enamel pattern.

The pika remain found in the Buena Pinta Cave, a 1.1 cm-long fragment of a right mandible, was recovered from grid unit L51 in Layer 3, at a depth of 210–220 cm. The fragment (inventory number MAR 2008/29/CBP/L51/3/175) is currently stored at the *Museo Arqueológico Regional de la Comunidad de Madrid* and consists of the corpus region of the mandible, preserved from the p4 alveolus through to the beginning of the ascending ramus (Fig 3). All the cheek-teeth except for p3 were standing in their corresponding alveoli. The length of the tooth row, measured on the occlusal view from the anterior part of p4 to the posterior part of m3, is 5.66 mm. The height of the mandibular corpus, taken under m2 is 4.98 mm, and its width at this same point is 2.88 mm. No further measurements could be taken on the mandible, due to its fragmentary state, but its maximal width at p4 has been estimated to be larger than 2.89 mm.

**Fig 4:**
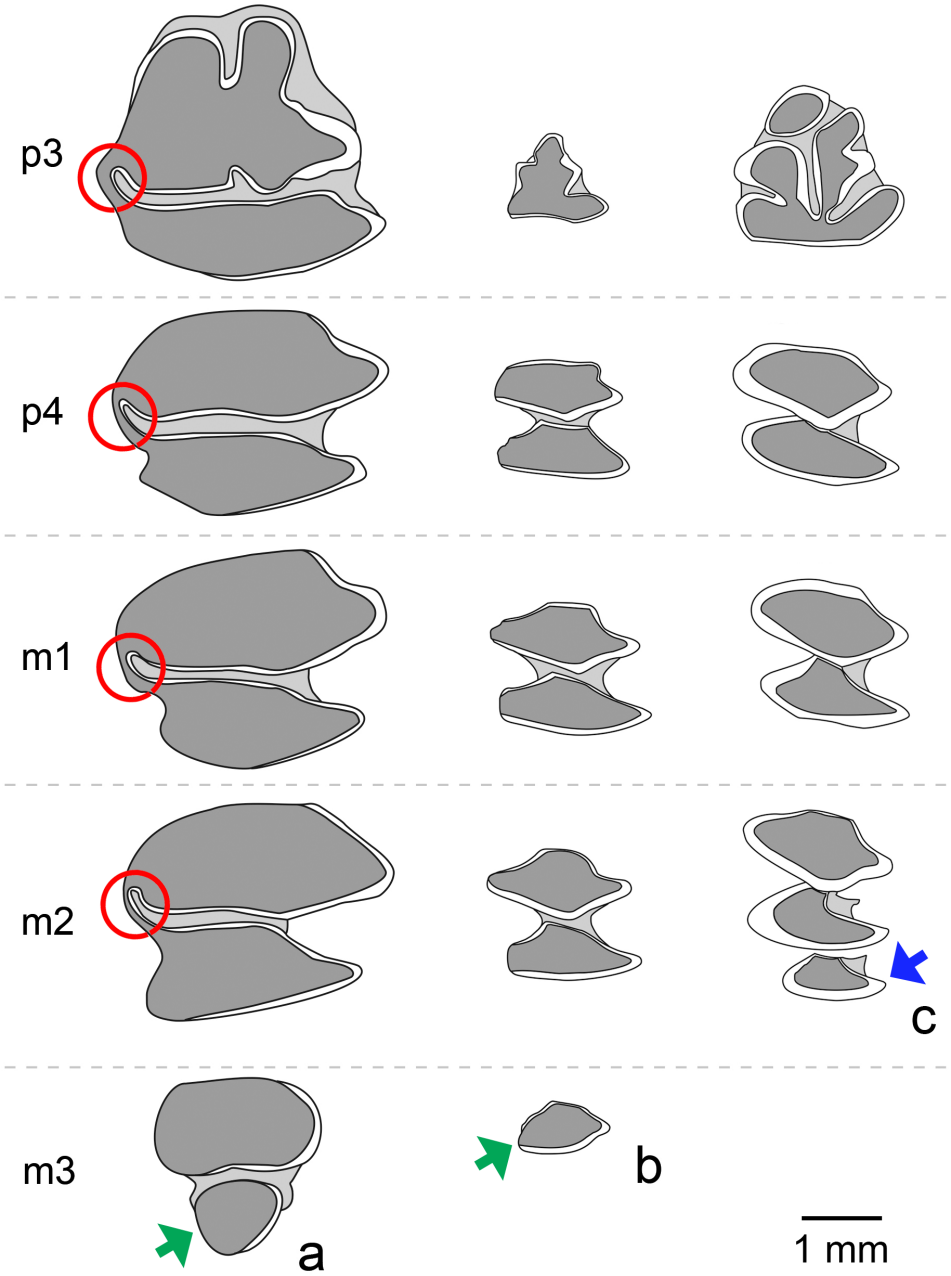
Comparison of the occlusal morphology of lower cheek-teeth of Leporidae, Ochotonidae, and Prolagidae. Leporidae (A) are represented by *Oryctolagus cuniculus* (redrawn from [87]), Ochotonidae (B) are represented by *Ochotona* sp. (redrawn from [88]), and Prolagidae (C) are represented by *Prolagus* sp. (redrawn from [25]). Red circles indicate dentine bridges connecting anterior and posterior lobes in Leporidae; green arrows indicate the presence of one or two lobes in m3 of Leporidae and Ochotonidae respectively; the blue arrow indicates the presence of three lobes in m2 of Prolagidae.

The discovered pika mandible is easy to distinguish from those of recent and Late Pleistocene lagomorphs of the Iberian Peninsula (Fig 4). Those of the Leporidae, represented by the rabbit (*Oryctolagus cuniculus*) and several hare species (*Lepus europaeus*, *Lepus granatensis*, *Lepus castroviejoi* and *Lepus timidus*) are considerably larger; the mandibles of early juveniles might be similar in size to that of an adult pika, but the presence of deciduous teeth or unworn permanent teeth in the former make their distinction simple. The lower dentition of leporids also differs from that of ochotonids by the m3 possessing two lobes rather than one (Fig 4). Moreover, in leporids, the two lobes of the lower molariform teeth (excluding m3) are connected by a lingual enamel bridge, whereas in ochotonids they are clearly separate.

The Prolagids are other lagomorphs that inhabited Iberia during the Pleistocene, represented by the single genus *Prolagus* (it should be noted that some authors consider them to belong to the family Ochotonidae (see [23,24], among others)). The latest records for *Prolagus* in continental Europe are those of the Cueva del Higuerón (Málaga, Spain) and Cova de Gracia (Barcelona, Spain) sites, both dated to the Middle Pleistocene [25]. Though the lower dental pattern in *Prolagus* is similar to that of *Ochotona*, three main differences can be seen: the presence of continuous enamel around both lobes of each tooth in *Prolagus* (see [26]), the absence of m3 in this same genus, and the presence of a third lobe in the m2 of *Prolagus* (instead of the typical two lobes of other lagomorphs).

The teeth preserved in the mandible from Buena Pinta Cave here described show the typical morphological pattern observed in ochotonids (see [26]), i.e., they have two lobes (the anteroflexid and postflexid lobes, respectively known as the trigonid and talonid lobes in some papers), except for m3, which has only one. In all teeth the enamel is thinner in the anterior part of the lobes, thicker in the posterior part, and absent on the lingual side. The same pattern can be seen in the *Ochotona* specimen discussed by Chaline [27] and in *O*. *pusilla* [28].

**Fig 5:**
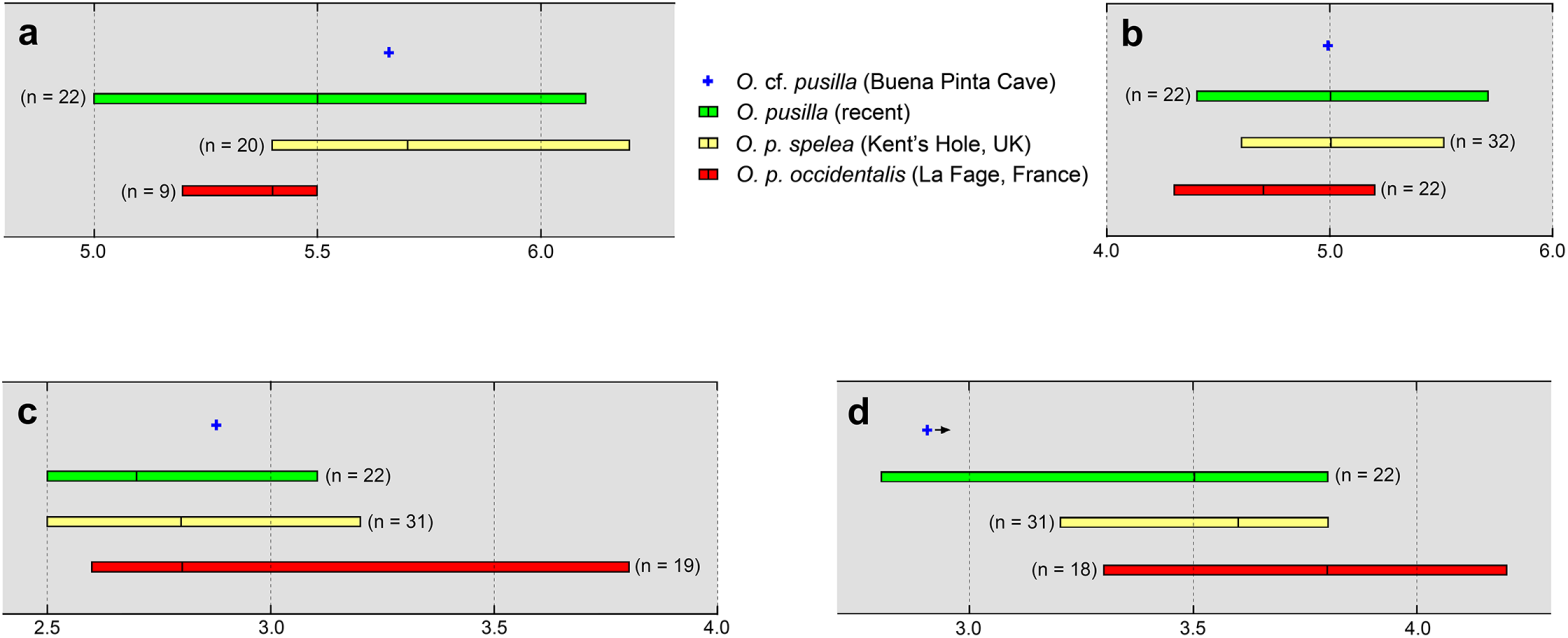
Comparison of the measurements of the jaw of *O*. cf. *pusilla* from Buena Pinta Cave with other recent and fossil samples of *O*. *pusilla*. (A) Coronar length of p4-m3. (B) Mandible height at m2. (C) Mandible width at m2. (D) Mandible width at p4. Data from the recent and fossil samples come from [12]. All the measurements are in millimetres.

Given the missing p3, an element of high diagnostic value in lagomorphs, the specific identification is more difficult to address. *O*. *pusilla* is the only pika species known to have inhabited western Europe during the Late Pleistocene [8]. The size of the mandible from Buena Pinta Cave falls within the range of variation observed in *O*. *pusilla*, both in recent and fossil material (Fig 5). Teeth other than the p3 are usually considered of low diagnostic value, and few biometric data are available for comparisons (Table 1). Measurements of the teeth of *O*. *pusilla* from Mamutowa Cave (Poland)[29], one of the few localities from which these data are known, are very similar to those from the mandible of Buena Pinta Cave. Thus, most of the measurements of the teeth of Buena Pinta Cave fall within the range of variation of the polish sample and only two parameters (length of m2 and width of m3) are slightly over their maximum values. However, these small differences may be due to the wide range of variation *O*. *pusilla* displayed during the Late Pleistocene in the length of the lower cheek teeth row as pointed out by Fladerer [30]. Compared to Early Pleistocene European fossil pikas (Table 1), the first and second lower molars of the mandible of Buena Pinta Cave are larger and fall out of the range of variation of both *O*. *polonica* from Zamkowa Dolna Cave (the type-locality of this species) [31] and *O*. *dehmi* from Schernfeld (the type-locality of this species) [32], although in the last case the sample is so small that little significance can be given to differences found when compared with other samples.

**Table 1:** Measurements of the lower teeth (p4 to m3) of *O*. *polonica* (Zamkowa Dolna Cave, Poland; Early Pleistocene [31]), *O*. *dehmi* (Schernfeld, Germany; Early Pleistocene [32]), *O*. *pusilla* (Mamutowa Cave, Poland; Late Pleistocene [29]) and *O*. cf. *pusilla* from Buena Pinta Cave. (tr: trigonid; tl: talonid).

	*O*. *polonica*				*O*. *dehmi*				*O*. *pusilla*				*O*. cf. *Pusilla*
	Zamkowa Dolna Cave				Schernfeld				Mamutowa Cave				Buena Pinta Cave
	n	mean	range	sd	n	mean	range	sd	n	mean	range	sd	
Lp4	4	1.35	1.14–1.44	0.14	-	-	-	-	8	1.36	1.28–1.43	0.06	1.34
Wp4tr	4	1.34	1.24–1.44	0.10	-	-	-	-	6	1.31	1.21–1.43	0.08	1.25
Wp4tl	4	1.51	1.37–1.59	0.10	-	-	-	-	7	1.55	1.47–1.66	0.07	1.50
Lm1	7	1.45	1.34–1.57	0.09	-	-	-	-	11	1.55	1.46–1.68	0.06	1.64
Wm1tr	6	1.44	1.21–1.60	0.14	-	-	-	-	11	1.60	1.43–1.71	0.09	1.63
Wm1tl	6	1.51	1.34–1.65	0.13	-	-	-	-	11	1.59	1.50–1.73	0.08	1.62
Lm2	7	1.41	1.29–1.52	0.09	2	1.55	1.55–1.55	0.00	11	1.54	1.43–1.64	0.06	1.67
Wm2tr	7	1.47	1.32–1.62	0.12	2	1.57	1.50–1.65	1.11	11	1.57	1.43–1.74	0.10	1.69
Wm2tl	6	1.42	1.27–1.55	0.12	-	-	-	-	10	1.49	1.31–1.58	0.09	1.52
Lm3	3	0.56	0.47–0.69	0.12	-	-	-	-	10	0.58	0.51–0.64	0.03	0.62
Wm3	3	1.06	0.95–1.25	0.17	-	-	-	-	9	1.01	0.87–1.11	0.07	1.14

Thus, despite the absence of the p3 in the Buena Pinta Cave mandible, it has been ascribed to *O*. cf. *pusilla* since *O*. *pusilla* is the only pika species known to have inhabited western Europe during the Late Pleistocene and because both the morphology and the size of the fossil mandible and teeth coincide with those of recent and fossil *O*. *pusilla*.

## Discussion: the Biogeographical Significance of the Presence of a Pika in the Buena Pinta Cave

**Fig 6:**
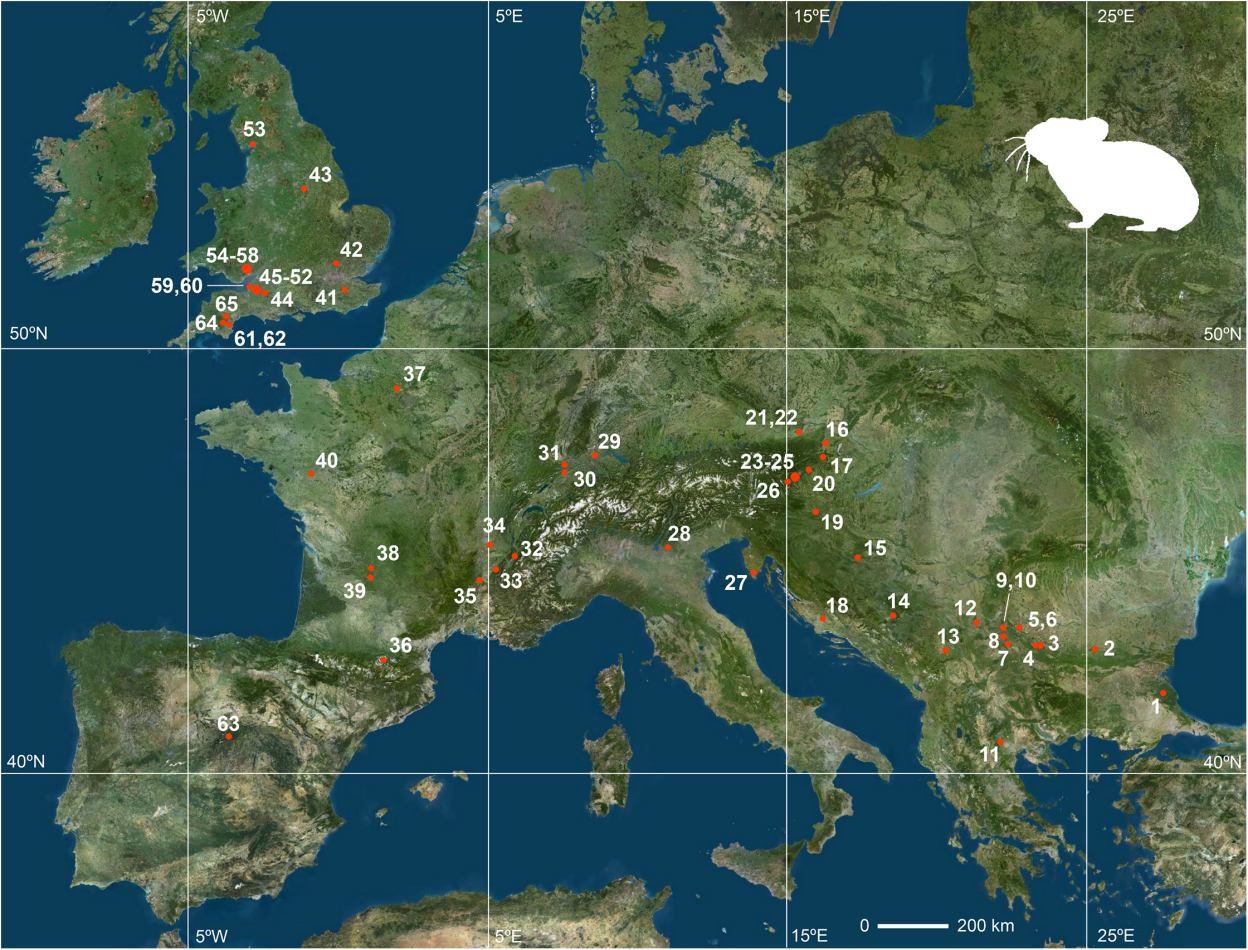
Map of Europe showing the southernmost and westernmost Late Pleistocene sites with remains of *Ochotona*. Sites are listed as in Table 2. Source of the satellite image: http://www.geoportail.gouv.fr/ (public domain).

Evidence exists that several species of pikas of the genus *Ochotona* were present in Europe since the Pliocene and, during the Pleistocene, usually coinciding with cold episodes [33]. However, records are quite rare until the Middle Pleistocene, though the largest number of records is for the Late Pleistocene. During this time, *O*. *pusilla* extended to occupy most of France (including its Atlantic coast) [8,10,34], and even southern and central Britain, although it never reached Ireland [11,35] (Fig 6). Towards the south, it reached the mountain ranges that form the northern limit of the Mediterranean peninsulas (Table 2). It arrived at the Balkan and Dinaric mountains of the Balkan Peninsula during the Late Pleistocene [10,36] and even reached the Pindus Mountains of Greece, where a record from Arnissa [37] is the southernmost for the species in Europe. In the Italian Peninsula it inhabited the northern and southern slopes and foothills of the Alps, the Riparo Tagliente site [38] providing the southernmost record for this region.

**Table 2:** List of southernmost and westernmost Late Pleistocene sites in Europe with remains of *Ochotona*. Sites are listed according to their longitude, from east to west.

Country	Site	Number in map	Reference	Original identification
Bulgaria	Mecha Dupka near Stoilovo	1	[57]	*Ochotona* cf. *pusilla*
	Bacho Kiro	2	[58]	*Ochotona* sp.
	Temnata Dupka Cave 16	3	[59]	*Ochotona pusilla*
	Mecha Dupka near Zhelen	4	[60]	*Ochotona* sp.
	Kozarnika	5	[61]	*Ochotona* sp.
	Redaka II	6	[62]	*Ochotona pusilla*
Serbia	Vrelska Cave	7	[63]	*Ochotona pusilla*
	Mala Balanica	8	[64]	*Ochotona pusilla*
	Baranica	9	[65]	*Ochotona pusilla*
	Vasiljska Cave	10	[66]	*Ochotona pusilla*
	Mirilovska Cave near Cuprija	12	[67]	*Ochotona pusilla*
	Smolucka Cave	13	[36]	*Ochotona pusilla*
Greece	Arnissa	11	[37]	*Ochotona pusilla*
Bosnia Herzegovina	Upper Bijambar Cave	14	[10]	*Ochotona pusilla*
Croatia	Kamenika	15	[68]	*Ochotona pusilla*
	Eastern Cave of Brina	18	[69]	*Ochotona pusilla*
	Vindija Cave	19	[69]	*Ochotona pusilla*
	Sandalja I	27	[69]	*Ochotona pusilla*
Austria	Allander Tropfsteinhöhle	16	[70]	*Ochotona pusilla*
	Mehlwurmhöhle	17	[71]	*Ochotona pusilla*
	Knochenhöhle near Kapellen	20	[72]	*Ochotona pusilla*
	Schusterlucke	21	[73]	*Lagomys pusillus fossilis*
	Willendorf	22	[74]	*Ochotona pusilla*
	Grosse Ofenberghöhle	23	[75]	*Ochotona pusilla*
	Grosse Badhöhle	24	[76]	*Ochotona pusilla*
	Grosse Peggauerwandhöhle	25	[77]	*Ochotona pusilla*
	Luegloch near Köflach	26	[78]	*Ochotona pusillus*
Italy	Riparo Tagliente	28	[37]	*Ochotona* sp. cf. *O*. *pusilla*
Switzerland	Schweizerbild	29	[79]	*Ochotona pusilla*
	Rislisberghöhle	30	[80]	*Ochotona pusilla*
	Ettingen	31	[81]	*Lagomys pusillus*
France	L’Abri de la Fru	32	[82]	*Ochotona pusilla*
	Le Campalou	33	[82]	*Ochotona pusilla*
	La Garenne	34	[82]	*Ochotona pusilla*
	Baume de Moula-Guercy	35	[40]	*Ochotona pusilla*
	Grotte de Bédeilhac	36	[44]	*Ochotona* sp.
	Brêche de Montmorency	37	[83]	*Lagomys pusillus*
	La Madeleine	38	[84]	*Lagomys pusillus*
	L’Abri Vaufrey	39	[85]	*Ochotona pusilla*
	Roc-en-Pail	40	[86]	*Ochotona pusilla*
Great Britain	Igtham Fissures	41	[11]	*Ochotona pusilla*
	Nazeing	42	[11]	*Ochotona pusilla*
	Robin Hood’s Cave	43	[11]	*Ochotona pusilla*
	Tom Tivey’s Hole	44	[11]	*Ochotona pusilla*
	Rowberrow Cavern	45	[11]	*Ochotona pusilla*
	Chelme’s Combe	46	[11]	*Ochotona pusilla*
	Cough’s Cave	47	[11]	*Ochotona pusilla*
	Sun Hole	48	[11]	*Ochotona pusilla*
	Soldier’s Hole	49	[11]	*Ochotona pusilla*
	Aveline’s Hole	50	[11]	*Ochotona pusilla*
	Bridged Pot	51	[11]	*Ochotona pusilla*
	Badger Hole	52	[11]	*Ochotona pusilla*
	Helsfell Cave	53	[11]	*Ochotona pusilla*
	Merlin’s Cave	54	[11]	*Ochotona pusilla*
	Great Doward Cave	55	[11]	*Ochotona pusilla*
	King Arthur’s Cave	56	[11]	*Ochotona pusilla*
	Symond’s Yat East	57	[11]	*Ochotona pusilla*
	Cavall’s Cave	58	[11]	*Ochotona pusilla*
	Wolf Den	59	[11]	*Ochotona pusilla*
	Hutton Cave	60	[11]	*Ochotona pusilla*
	Wolf’s Cave	61	[11]	*Ochotona pusilla*
	Happaway Cave	62	[11]	*Ochotona pusilla*
	Broken Cavern	64	[11]	*Ochotona pusilla*
	Cow Cave	65	[11]	*Ochotona pusilla*
Spain	Cueva de la Buena Pinta	63	Current paper	*Ochotona* cf.*pusilla*

The Buena Pinta Cave finding is the first record for a pika in Iberia. As such, it marks a considerable increase in the southwestern European range of the genus *Ochotona* during the Pleistocene. Buena Pinta Cave is located at the centre of this Peninsula, over 500 km from the until-now most southwestern record for the species. There is good evidence that pikas (*O*. *pusilla*) were present in southern France at least from the middle of the Middle Pleistocene (records are known from Layers P, L and K of the Caune de l’Arago site dating to MIS14-MIS12 [39,40]) until the end of this period (Layer XVIII of the Baume Moula-Guercy dating to MIS6 [41]) and throughout the Late Pleistocene from the end of MIS5 (with records from Portel-Ouest [42,43]) to the Late Pleistocene–Holocene transition (from Pont d’Ambon [42]). The French records closest to the Buena Pinta Cave are from Bédeilhac [44] and Portel-Ouest [43] (end of the Late Pleistocene) on the northern slopes of the Pyrenees. Despite the proximity of these records to the Iberian Peninsula, it is remarkable the complete absence of pikas in its northern half. Although numerous Late Pleistocene sites in the Cantabrian and Pyrenean region have extended stratigraphic sequences, and detailed studies of their small vertebrate assemblages have been conducted (see reviews in [22,45,46]), no pika remains have ever been reported. Certainly, these sites contain the remains of other immigrant species from northeastern Europe and central Asia such as *Microtus gregalis*, *Microtus oeconomus*, *Sicista betulina* and *Lepus timidus* [22,47,48,49], but they provide no evidence that pikas came with them. However, a careful review of the lagomorph material from the northern sites of the Iberian Peninsula would be worthwhile since pikas may have been taken for juvenile rabbits. Rabbits (*Oryctolagus cuniculus*) are very common in Iberian sites and well represented throughout the Pleistocene, and though the morphological and biometric differences between adult rabbits and pikas are ample, juvenile rabbits overlap in size with adult pikas, thus enabling misidentification.

At the Buena Pinta Cave, the intensive effort undertaken in sampling the small vertebrates of the site led to the recovery of more than 250,000 small vertebrate fossils over 11 excavation campaigns. This allowed even the least abundant taxa in the material to be detected. The finding of the single fossil of *O*. cf. *pusilla* was the consequence of the careful analysis of the several thousand lagomorph remains (mostly of *O*. *cuniculus*) obtained.

**Fig 7:**
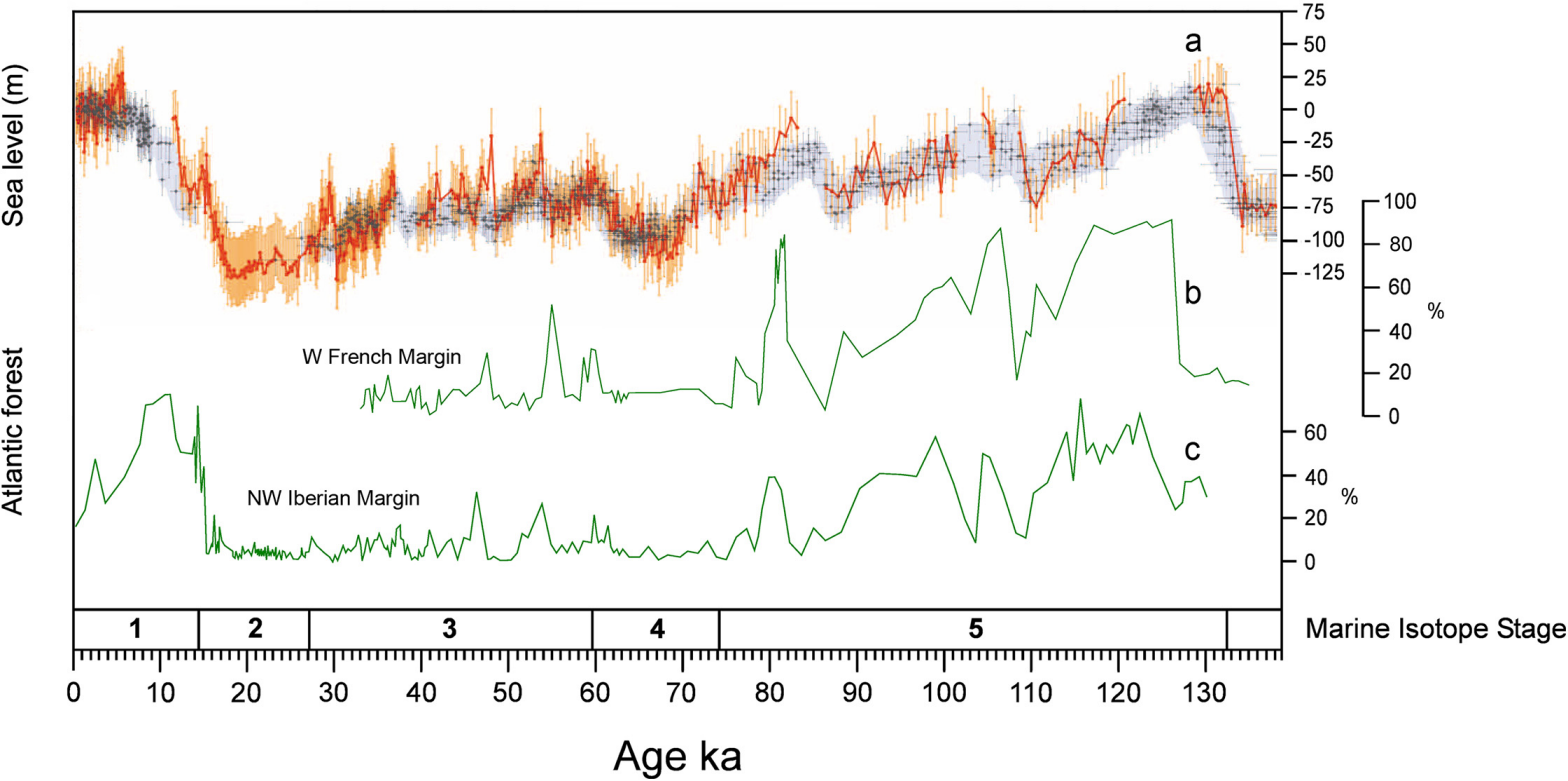
Changes of sea level (RSL) values in the Mediterranean, and Atlantic forest development in southern France and north of the Iberian Peninsula during the last 135 ka. (A) RSL at Gibraltar; (B) Fluctuations of Atlantic forest development in southwestern France based on pollen record from deep-sea core MD04-2845 (136–30 ka), at 45°N, 05°W; (C) Fluctuations of Atlantic forest development in the nortwest of the Iberian Peninsula based on the pollen record from deep-sea cores MD99-2331 (131–14 ka) and MD03-2697 (14–0 ka) at 42°N, 09°W. Fig 7A reprinted from [50], with permission from Elsevier. Fig 7B and 7C reprinted from [51] with permission from Macmillan Publishers Ltd.

The distribution pattern of pikas in southern Europe during the Late Pleistocene suggests the continent’s alpine mountain ranges acted as dispersal barriers; only on a few occasions did pikas succeed in crossing or circumventing them to reach the peninsulas of the Mediterranean. Fig 6 shows that most of the southernmost records of *Ochotona* in the continent during the Late Pleistocene are located on the northern slopes of the alpine mountain ranges, and only some of them (Arnissa in Greece, Eastern Cave of Brina and Sandalja in Croatia, Riparo Tagliente in Italy and Buena Pinta Cave in Spain) are on their southern slopes or more to the south. The role these mountain ranges had as geographic barriers was probably not strictly due to the mountains themselves, but to the forests that developed around them or on their slopes, that acted as effective ecological barriers to species adapted to open steppe landscapes. During the coldest periods of the Late Pleistocene, however, the combined effect of the reduction of forest cover and the emersion of large areas of the continental shelf due to the drop in sea level, provided steppic small mammals such as the steppe pika (the only pika species known to have inhabited western Europe during the Late Pleistocene) a unique opportunity to avoid these habitats and enter into the mediterranean peninsulas. Concerning the Pyrenees, Fig 7 compares the changes inferred for the sea level of the Mediterranean during the Late Pleistocene [50] and in atlantic forests development of in the northern part of the Iberian Peninsula as inferred from the pollen record from three different deep-sea cores (MD04-2845 drilled in the Bay of Biscay; MD95-2331 and MD03-2697 drilled in the northwest Iberian margin) [51]. These two conditions (the reduction of the importance of Atlantic forests and low sea levels) were particularly pronounced at the end of MIS6 and from the beginning of MIS4 until the end of MIS2 (though important fluctuations occurred during MIS3). It is during MIS6 when immigrant taxa from the northeast of Europe are first recorded in the Iberian Peninsula. Until then, and therefore for the most part of the Middle Pleistocene, there is no record in the Iberian Peninsula of these cold environment, northeastern European small mammal species (*Microtus oeconomus*, *Microtus gregalis*, *Chionomys nivalis*, *Spermophilus citellus*, *Spermophilus major*, *Sicista betulina*, *Lepus timidus*, *Ochotona pusilla*). The earliest record of this group of species in Iberia is that of *M*. *oeconomus* and *Sicista betulina* from MIS6 lowermost layer in Lezetxiki II [52]. But it is from MIS4 onwards when more of these taxa start to become frequent and varied in Iberian sites. For instance, to this period belong the earliest records of *C*. *nivalis* in the Iberian Peninsula (layers X and XI of Cueva de la Carihuela [53]; south sector in Cueva del Camino [15]); or those of *M*. *gregali*s and the single record of *O*. cf. *pusilla* referred to in this paper. Records become increasingly abundant in MIS3 and MIS2 [46]. It is unclear why the entry of these species was delayed until the Late Pleistocene and did not happen in any of the previous cold episodes of the Middle Pleistocene during which some of them had already reached southern France. Some authors [54] have suggested that the north of the Iberian Peninsula during the Middle Pleistocene (500–200 ka) was characterized by highly stable ecological conditions during which no relevant changes took place in vegetation landscapes, favouring that the Pyrenees acted then as an effective ecological barrier.

Layer 3 of the Buena Pinta Cave, where the pika mandible was found, was deposited some 63.4±5.5 ka ago. Thus it does not belong to MIS2, i.e., the period in which mammalian species well adapted to cold climates were most common in the Iberian Peninsula [55]. Rather, it belongs to an earlier moment within MIS4 or the beginning of MIS3. In this layer, it is accompanied by other cold-adapted small mammals such as *M*. *oeconomus*, *M*. *gregalis* or *Ma*. *marmota*. The cave’s records for these species are either the southernmost or among the most southerly for Late Pleistocene Europe. The occurrence of a pika together with other cold-adapted small mammals suggests the development of steppe landscapes in the interior of the Iberian Peninsula during the Late Pleistocene. This agrees with the interpretations of pollen analyses for cave sites of similar age in central Spain e.g., Cueva de los Torrejones [56] (although the considerably smaller microvertebrate sample size in this site means cold-adapted small mammals are less well represented).

## Conclusions

The present pika mandible fragment found in Layer 3 (age 63.4±5.5 ka) of the Buena Pinta Cave increases by 500 km the known southwestern limit of *Ochotona* in Europe during the Pleistocene. An important diagnostic dental element in ochotonids, p3, is missing from the fossil found at Buena Pinta Cave, but the morphology and size of its remaining features are all in line with those of *O*. *pusilla*, the only pika species known to have inhabited Europe during the Late Pleistocene. The fossil is therefore ascribed to *O*. cf. *pusilla*. The absence of pika records from northern Iberia may be a consequence of insufficiently thorough sampling of small mammal assemblages, or of misidentification of its infrequent remains for those of rabbits (which are very common). The co-occurrence of the pika with other cold-adapted species in Layer 3 of the Buena Pinta Cave supports the idea that, during the middle part of the Late Pleistocene, steppe landscapes were present in the interior of the Iberian Peninsula.
